# Archetype relational mapping - a practical openEHR persistence solution

**DOI:** 10.1186/s12911-015-0212-0

**Published:** 2015-11-05

**Authors:** Li Wang, Lingtong Min, Rui Wang, Xudong Lu, Huilong Duan

**Affiliations:** College of Biomedical Engineering and Instrument Science, Zhejiang University, Room 512, Zhouyiqing Building, Zhejiang University, 38 Zheda Road, Hangzhou, Zhejiang China; Department of Information Technology, Shanxi Dayi Hospital, Taiyuan, Shanxi China

**Keywords:** Archetype relational mapping, Archetype, OpenEHR, Relational database, Data persistence

## Abstract

**Background:**

One of the primary obstacles to the widespread adoption of openEHR methodology is the lack of practical persistence solutions for future-proof electronic health record (EHR) systems as described by the openEHR specifications. This paper presents an archetype relational mapping (ARM) persistence solution for the archetype-based EHR systems to support healthcare delivery in the clinical environment.

**Methods:**

First, the data requirements of the EHR systems are analysed and organized into archetype-friendly concepts. The Clinical Knowledge Manager (CKM) is queried for matching archetypes; when necessary, new archetypes are developed to reflect concepts that are not encompassed by existing archetypes. Next, a template is designed for each archetype to apply constraints related to the local EHR context. Finally, a set of rules is designed to map the archetypes to data tables and provide data persistence based on the relational database.

**Results:**

A comparison study was conducted to investigate the differences among the conventional database of an EHR system from a tertiary Class A hospital in China, the generated ARM database, and the Node + Path database. Five data-retrieving tests were designed based on clinical workflow to retrieve exams and laboratory tests. Additionally, two patient-searching tests were designed to identify patients who satisfy certain criteria. The ARM database achieved better performance than the conventional database in three of the five data-retrieving tests, but was less efficient in the remaining two tests. The time difference of query executions conducted by the ARM database and the conventional database is less than 130 %. The ARM database was approximately 6–50 times more efficient than the conventional database in the patient-searching tests, while the Node + Path database requires far more time than the other two databases to execute both the data-retrieving and the patient-searching tests.

**Conclusions:**

The ARM approach is capable of generating relational databases using archetypes and templates for archetype-based EHR systems, thus successfully adapting to changes in data requirements. ARM performance is similar to that of conventionally-designed EHR systems, and can be applied in a practical clinical environment. System components such as ARM can greatly facilitate the adoption of openEHR architecture within EHR systems.

## Background

Currently, an electronic health record (EHR) system is essential to support clinical practice with information technology in healthcare environments. EHR is a repository of information regarding the health status of a subject of care in computer processable form [1]. However, healthcare data is generally too complicated, flexible, and changeable to capture a universal, comprehensive and stable schema of information, which is the foundation of the entire EHR architecture. Highly-specialized and complex EHR systems cannot acclimate to the evolution of healthcare data requirements, which require EHR systems to embrace a dynamic, state-of-the-art, rapidly evolving information infrastructure [2]. In order to protect EHR systems from changes in the healthcare domain, openEHR [3] has published a series of specifications to guide the development of future-proof EHR systems. Solutions span from information modelling to system architecture, to meet the continually evolving needs of EHR systems.

The concept of openEHR focuses on systems and tools necessary to the computation of complex and constantly evolving health information at a semantic level, according to the following three paradigms: separation of information models, domain content models, and terminologies; separation of responsibilities; and separation of viewpoints [4]. Among these three paradigms, the separation of information models, domain content models and terminologies promotes a significant shift from the single-level modelling approach of information system development to a two-level modelling approach. In the single-level modelling approach, domain concepts that are processed by the EHR system are hard-coded directly into the application and database models. In two-level modelling, the semantics of information and knowledge are separated into a small, comprehensible, non-volatile reference model (RM), which is used to build information systems and knowledge models; archetypes are used as formalisms and structures to express numerous and volatile domain concepts [5].

The RM represents the general features of health record components, their method of organization, and necessary contextual information to satisfy both the ethical and legal requirements of the health record. The RM encompasses the stable features of the health record by defining the set of classes that composes the blocks which in turn constitute the record. Archetypes define entire, coherent informational concepts from the clinical domain [6]. An archetype is a hierarchical combination of components from the RM with available restrictions placed on names, possible data types, default values, cardinality, etc. These structures, although sufficiently stable, may be modified or replaced by others as clinical practice progresses and evolves [7]. Archetypes are deployed at runtime via templates that specify particular groups of archetypes to be used for a particular purpose, often corresponding to a screen form. A template is a specification that creates a tree structure of one or more archetypes, and each constraining instance of various RM types such as composition, section, entry subtypes, etc. Templates typically closely correspond to screen forms, printed reports, and generally complete information to be captured or sent at the application level; they may therefore be used to define message content [4].

By utilizing a two-level modelling approach, EHR systems can be built on a stable RM as a general framework, and thus use archetypes as the domain information model to achieve greater flexibility and stability, particularly in situations in which the domain concepts are vast in number, have complex relationships, and evolve continuously. The responsibilities of specialists from the information technology domain and the healthcare domain are disengaged: developers focus only on the technical components of EHR systems, while specialists develop the structural model based on domain concepts and archetypes. This enables domain specialists to participate directly in the production of the artefacts that are interpreted by EHR systems, to organize and present healthcare information, and to control the EHR system without intervention from the system supplier or re-programming.

Syntactic and semantic interoperability between different EHR systems is facilitated by the use of archetypes. However, agreement between data content and the information models is necessary for real integration and semantic interoperability [8]. In the single-level modelling approach, the domain concepts are implicitly contained within the EHR systems. No consensus healthcare domain models exist in healthcare domain models to which EHR systems may conform; each system may model different aspects and granularity levels of domain concepts, resulting in heterogeneity. In the two-level modelling approach, the RM ensures that EHR systems can always send information to other systems and receive readable information in return, thus ensuring data interoperability. Archetypes can be used as the common knowledge repository to share evolving clinical information that can be processed by the receiving systems, thus enabling semantic interoperability [9]. The openEHR system maintains Clinical Knowledge Manager (CKM) as an official archetype repository to support the governance of international domain knowledge.

Specifications of openEHR can be used to create and sustain a flexible EHR ecosystem that consists of may integrated services, which is too complex to be accurately processed by single applications [10]. The general architectural approach can be considered as five layers: persistence (data storage and retrieval), back-end services (including EHR, demographics, terminology, archetypes, security, record location, etc.), virtual EHR (a coherent set of APIs to the various back-end services and an archetype-and-template-enabled kernel responsible for creating and processing archetype-enabled data), application logic (user applications or another service, such as a query engine), and presentation (the graphical interface of the application) [4]. The archetypes share the openEHR innovation of adaptability because they are external to the software, while key components of the software are derived from the archetypes [11]. Much current research has been devoted to the use of archetypes to drive the persistence, accessibility, and presentation of healthcare information systems [12–16].

Most EHR systems in the healthcare field are built according to the single-level modelling approach, despite the many advantages of two-level modelling. One of the primary reasons is that the persistence layer is inadequate to meet the requirements of clinical practice. As the foundation of EHR systems, the persistence layer determines the EHR system architecture, and thus also function as a performance bottleneck. The openEHR system promotes a Node + Path persistence solution that serializes sub-trees of fine-grained data into blobs or strings based on the object or relational systems [17]. The data can be serialized according to different granularity levels, from top-level information objects to the lowest leaf nodes. In essence, the Node + Path solution is an entity-attribute-value (EAV) approach, which takes advantage of the semantic paths in openEHR data to improve the serialized-blob design. The greatest advantages of the Node + Path approach are flexibility and simplicity; all data nodes are serialized, and their paths are recorded adjacently in a two-column table of < node path, serialized node value>. However, the simplicity of the data storage structure induces complex data retrieval logic, which strains the performance of data insertion and query, which requires flexibility [18]. Some researchers have reported similar performances in the processing of entity-centered queries by conventional and EAV models, but that EAV models were approximately 3–5 times less efficient in the processing of attribute-centered queries [19]. Evaluations of persistence solutions using an XML database also indicates that XML databases were considerably slower and required much more space than the relational database [20]. There has also been similar research into the data persistence of alternative approaches based on two-level modelling, such as EN13606 [21] or HL7 Version 3 [22]. In a proof-of-concept work of EN13606 [7], the data storage was developed by applying Object Relational Mapping (ORM) [23] to the RM of EN13606; this approach was investigated by the authors in earlier work [24]. The deep inheritance and complicated relationships of the EN13606 RM induces excessive JOIN operation during data query. Additionally, classes near the top hierarchy become heavily overloaded with data. For example, DATA_VALUE is the basic class of all data types, and contains the common attributes of all instances of all data classes, but it is unable to operate in real time [7]. IBM Clinical Genomics medical research developed a hybrid data model based on the HL7 Version 3 Reference Information Model (RIM) [25]. The hybrid data model combines elements of both the ORM and EAV approaches, which is well-suited to the sparse and flexible data of a medical research data warehouse. However, it demonstrates similar problems to other ORM and EAV approaches in that it does not improve the performance enough to support effective clinical transactions.

The relational database is still of primary importance to the data persistence of EHR systems, and its excellent performance has already been well proved in many successful EHR projects and widely-accepted EHR productions. The primary challenge to the application of a two-level modelling approach to a relational database is that the domain information model is hard-wired into the database model; when the domain information model changes, the relational database must be redesigned in order to facilitate the new domain information model. There are two approaches to support the adaptation of the persistence layer to changes in archetypes. One approach is to use a general data storage structure that is independent of archetypes, such as Node + Path, which has been well-investigated. The other approach is to generate a database model to design a persistence layer driven by archetypes.

This work presents an archetype relational mapping (ARM) persistence solution based on a relational database that can achieve similar performance to the conventional database in practical clinical environments. This work extends the basic ARM method introduced in a previous proof-of-concept work [24] by providing a more sophisticated mapping approach based on templates and archetypes in order to map archetypes to relational tables. Performance optimization rules and details are then provided. First, the ARM approach is introduced in detail, including archetype modelling, template definition, and mapping rules. Then, the ARM is applied to the EHR data requirements of a tertiary hospital in China. A comparison of the generated ARM database, the conventional database deployed in the hospital, and the openEHR official Node + Path database is conducted. After analysis, the challenges encountered during ARM development are discussed, and conclusions are provided.

## Methods

An underpinning principle of openEHR is the use of archetypes and templates, which represent formal models of domain content that are used to control data structure and content during creation, modification, and querying [26]. The ARM approach employs archetypes and templates to generate a persistence model and provide data access based on a relational database. The ARM is intended to fulfill several functionalities:

1. Effective adaptation to changes in archetypes. Archetypes reflect the changing realities of EHR, and existing archetypes are updated over time. Archetype changes result in several challenges to ARM, such as the application of new archetypes to the relational database, and the possibility of incompatible versions among archetypes, among other potential challenges.
2. Generation of customized persistence models for various EHR requirements. Archetypes define widely reusable components of information, and templates encapsulate the local usage of archetypes and relevant preferences. In order to apply to the local EHR context, ARM employs templates in order to customize the data persistence model. There are three steps to employ ARM in the implementation of the persistence layer: archetype modelling, template definition, and database mapping.

### Archetype modelling

Archetype modelling selects and expands existing archetypes from the public archetype repository, or defines new ones in order to meet all data requirements. Archetype modelling has been well-developed, and widely applied in previous studies [27–29]. First, all data items must be determined by collecting and analysing the data requirements in detail, such as dataset specifications or database schemas. The data items must then be merged into coherent and meaningful clinical concepts. Then, existing archetypes must be reused as much as possible. Keywords are used to search the CKM for matching archetypes, including concept name and core data items. Concepts are identified as fully covered by archetypes, partially covered by archetypes, and/or not covered by archetypes. The archetype will be directly reused if it fully covers a concept, extended if it partially covers a concept, and new archetypes are designed for concepts with no existing archetypes.

### Template definition

Archetypes describing the general healthcare concepts are adapted to local EHR data requirements by template definition design templates. One corresponding template is designed for each archetype in order to add constraints, such as local optionality, archetype chaining, tightened constraints, default values [26], and ARM constrains. ARM constrains attempt to achieve better performance by aligning the concept-focused archetype model and the data-focused relational model. The ARM constraints are designed as follows:

1. Assign identification data item. An identification data item is used to uniquely identify instances of each archetype; it can represent any basic data type (Table 1) and has an occurrence of 0..1 or 1..1. Only one identification data item can exist for each archetype.

**Table 1:** Archetype basic data types and mapping rules

Data type	Field	Field data type	SQL type
CodePhrase	codeString	String	NVARCHAR
DvBoolean	value	Boolean	INTEGER
DvCodedText	definingCode	CodePhrase	#
DvCount	magnitude	Integer	INTEGER
DvDateTime	value	String	NVARCHAR
DvEHRURI	value	URI	NVARCHAR
DvIdentifier	id	String	NVARCHAR
DvMultimedia	uri	DvURI	#
DvProportion	precision	Integer	INTEGER
DvQuantity	magnitude	Double	FLOAT
	units	String	NVARCHAR
DvText	value	String	NVARCHAR
DvURI	value	URI	NVARCHAR
GenericID	value	String	NVARCHAR
	name	String	NVARCHAR
Link	target	DvEHRURI	#
2. Assign query data item. Some data items may always be used as query conditions, particularly those with identical characteristics to the identification data item. These types of data items can be categorized as query data items, and an archetype may have multiple query data items to facilitate the data query. Collection data structures such as CLUSTER, ITEM_TREE, ITEM_LIST, and archetype slots cannot be used as identification data items or query data items. As basic units of internal structures of archetypes, collection data structures group related data items, and can thus be viewed as embedded archetypes with their own identification data items and query data items.
3. Define mappings between generalized archetypes and specialized archetypes to facilitate data query. If a domain contains many concepts and data items with similar structures, a general concept with a data item can be used to store the name and all fields related to these concepts. It is impossible to simultaneously define the vast number of concepts included in EHR systems. Additionally, the definitions are difficult to maintain due to the continuous development of new concepts. Archetypes can clearly represent the general concept and specific concepts by specialization. The name and fields of data items in specialized archetypes are mapped to a subset of fields of the corresponding data item in the generalized archetype. Then, the data stored as a generalized archetype instance can be queried with specialized archetypes, and vice versa, using the mappings.

### Database mapping

Database mapping generates a relational database schema in order to automatically persist the data represented by archetype instances into relational databases using archetypes, templates, and ARM constraints. A set of mapping rules is designed as follows:

1. Map each archetype to a table. According to the archetype semantic relationship specified in openEHR specifications (Table 2) [30], new and old version of the same archetype can be organized into a single data table.

**Table 2:** Semantical relationships of archetypes

Relationship	Modification	Compatibility
Revision	Modify description part	Ensure backward compatibility
	Expand attributes, range of value sets, terminology	Data created by pre-revised archetype is compatible with the revised version
Specialization	Strengthen the constraints	Ensure the new specialized archetype must create data that conforms to the parent
	Redefine and add nodes	
	The range of value sets and semantics of nodes conform to the previous archetype	
New version	Change mandatory item to optional	Modifications are incompatible with the previous archetype
	Adjust value range or coded term set
2. Map the basic data items represented by the archetype basic data type. If the upper bound of the data item occurrence is 1, this indicates a single occurrence data item which can be mapped as a common column. If the upper bound of the data item occurrence is *, this indicates a multiple occurrence data item which is mapped into a standalone table with two columns: one is a foreign key column referring to the identification data item of the current archetype, and the other is a common column mapped from the data item. Table 1 lists the archetype basic data types utilized in ARM and their corresponding mapping rules. For fields with non-preliminary types in each archetype basic data type, the corresponding SQL type is noted as “#” and the mappings must be referenced in Table 1.
3. Constrain the identification data item as the key column. Unique and clustered index constraints are mapped from the identification data item, and added to the column. If there is no identification data item in an archetype, a data item named “id” is generated and used as the identification data item to identify records in the database table; the generated “id” is invisible and cannot be accessed using the archetype.
4. Constrain the query data item as an indexed column. The non-clustered index constraints mapped from query data items are added to columns in order to accelerate data query.
5. Map archetype slots. If the upper bound of the archetype slot occurrence is *, this indicates that the current archetype and the target archetype exhibit a one-to-many relationship, which is mapped as a foreign key column in the target archetype table, referring to the identification data item of the current archetype. If the upper bound of the archetype slot occurrence is 1, this indicates that the current archetype and the target archetype exhibit a one-to-one relationship, so the data items of the target archetype are embedded into the table of the current archetype, thus embedding the target archetype into the current archetype.
6. Map collection data items according to collection data structure. If the upper bound of the collection data item occurrence is 1, this indicates a single occurrence collection data item, so the data items contained in this collection data item can be mapped into the table of the current archetype, and can be viewed as flattened. If the upper bound of the collection data item occurrence is *, this indicates a multiple occurrence collection data item that is mapped into a standalone table with one foreign key column, referring to the identification data item of the current archetype.
7. Propagate query data items. An efficient method to reduce the recursive level deep in the archetype hierarchy tree when querying the leaf archetype is to store the most frequently queried data items of the ancestors in the descendants. For multiple-occurrence archetype slots and collection data items, the query data items in the current archetype can be mapped identically to the identification data item, into the target archetype data table of the archetype slot or the standalone data table of the collection data items as foreign key columns.
8. Naming. Naming rules vary slightly, because each relational database product, such as Microsoft SQL Server or Oracle, has unique restrictions for naming tables and columns. The general principles are as follows. The archetype name is used as the table name for tables mapped from archetypes. The archetype name concatenated with the data item name is used as the table name for tables mapped from collection data items and multiple occurrence data items; the version portion of the archetype name should be removed, since all versions of an archetype are mapped into the same database table. The path of the data item concatenated with the field name is used as the column name for a column mapped from a data item field; the path of the data item and the column name are unique, but the human readability of the path is poor. One alternative to achieve better readability is to use the textual name provided within the archetype ontology section of the data item rather than the path. However, the uniqueness of the textual name provided within the archetype ontology section of the data item is guaranteed; if the generated names for the table and column are so long as to violate the naming restrictions of the relational database products, they should be shortened in a consistent manner in order to remain unique.

### Database comparison

A performance comparison of the ARM approach and an EHR system used in real clinical practice in conducted. The compared EHR system has been deployed in a tertiary class A Chinese for three years. Several legacy systems have been integrated into the EHR system, including HMIS (hospital management information system), LIS (laboratory information system), RIS (radiology information system), PACS (picture archiving and communication system), PMS (pharmacy management system), and OMS (operation management system). Two information systems, CPOE (Computerized Physician Order Entry) and IV (Integrated Viewer), support the order-centered clinical workflow for all clinicians from all departments within the hospital. The IV information system allows clinicians to view the demographic, imaging examination, and laboratory test data of a patient scattered in heterogeneous silo systems in one application, rather than being forced to access different patient data in each corresponding system. The system represents a typical centralized data-reporting application in a hospital that presents patient information from all examination departments and encompasses most EHR data, thus serving as an ideal candidate against which to apply the ARM approach and conduct the comparison.

### Ethics

This study was approved by Review Board of Shanxi Dayi Hospital under project 2012AA02A601. A database specialist from the hospital’s information technology department exported and de-identified the necessary data of IV database.

## Results

### ARM mapping

**Fig. 1:**
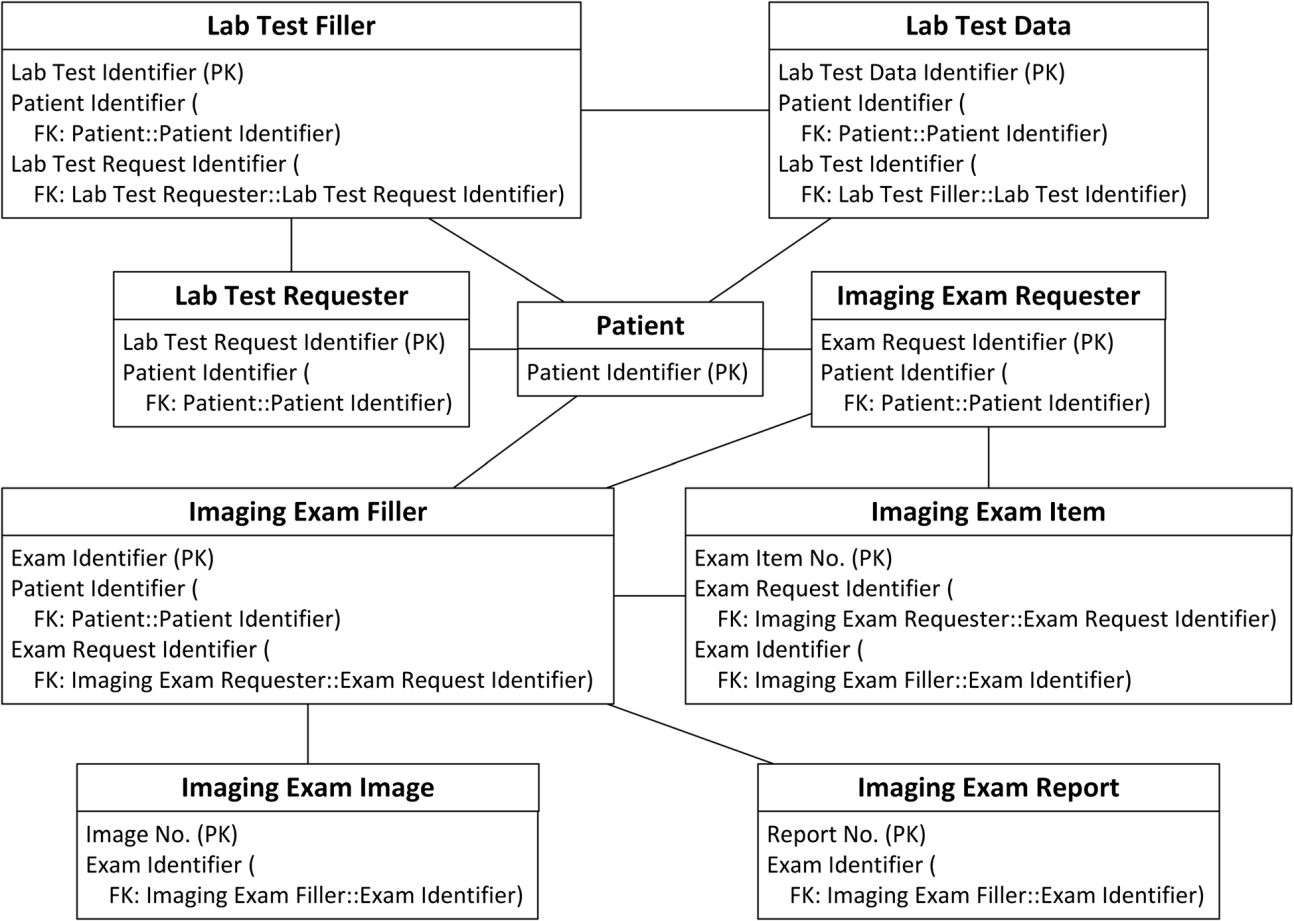
IV concepts overview. In all figures, PK stands for primary key. FK stands for foreign key, and indicates the data item which current data item relates to. SLOT indicates target archetype which conform to current data item. Solid line indicates foreign key relationship between archetypes. Dash line indicates composition relationship through slot between archetypes. IDI stands for identification data item. QDI stands for query data item. CI stands for clustered indexed. NCI stands for non-clustered indexed. Data items in italic type are not covered by archetypes

The schema of the IV database has been analysed in detail and extracted into concepts. Fig. 1 depicts the overview of the IV concepts and their relationships.

**Fig. 2:**
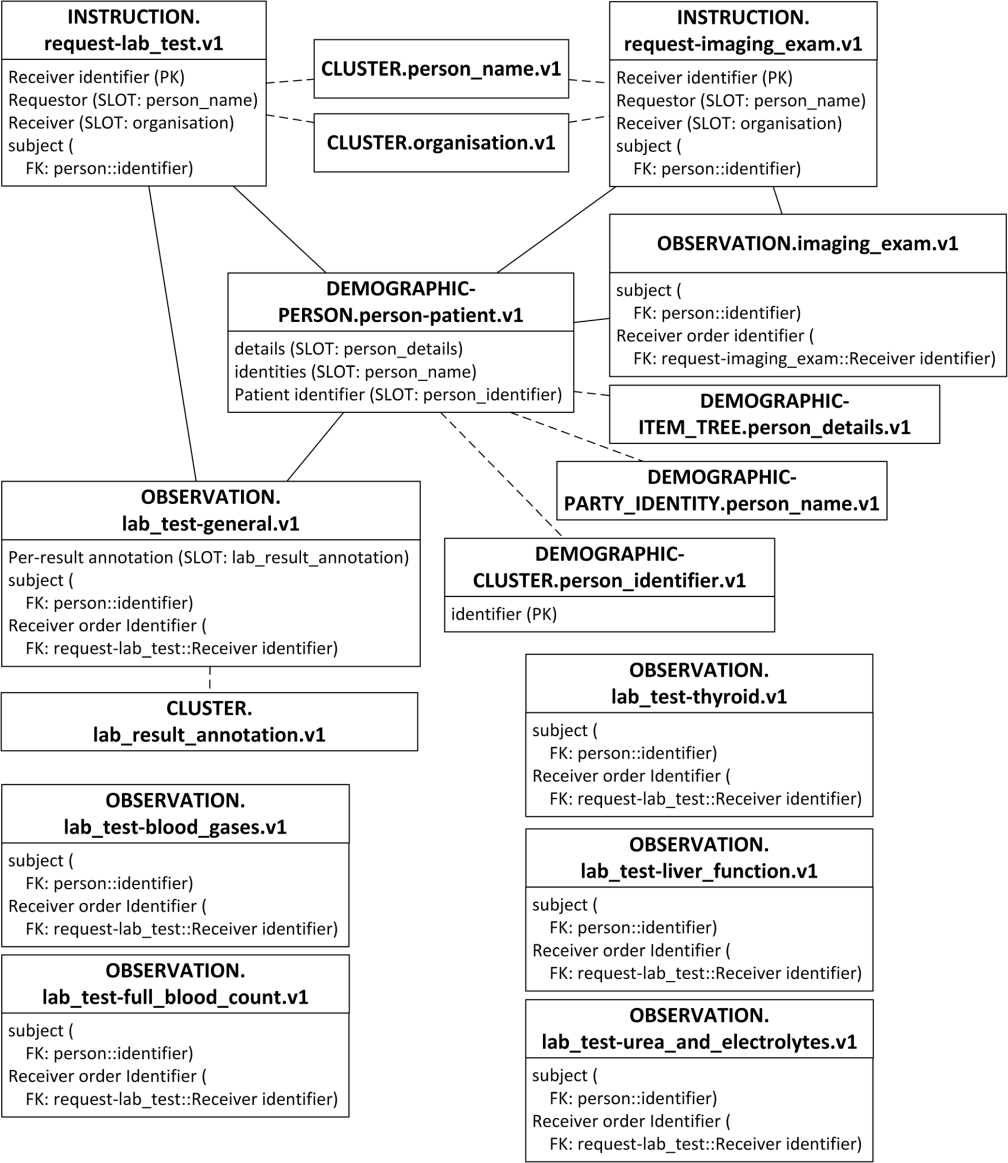
Archetypes overview. In all figures, PK stands for primary key. FK stands for foreign key, and indicates the data item which current data item relates to. SLOT indicates target archetype which conform to current data item. Solid line indicates foreign key relationship between archetypes. Dash line indicates composition relationship through slot between archetypes. IDI stands for identification data item. QDI stands for query data item. CI stands for clustered indexed. NCI stands for non-clustered indexed. Data items in italic type are not covered by archetypes

The primary purpose of this investigation is to explore the performance of the ARM approach. Existing matching archetypes in CKM are selected without modification to facilitate clear interpretation of comparisons. A total of 17 archetypes are selected to encompass the IV concepts. Fig. 2 depicts an overview of the selected archetypes and their relationships.

A total of 16 are existing archetypes:

- openEHR-DEMOGRAPHIC-PERSON.person-patient.v1
- openEHR-DEMOGRAPHIC-ITEM_TREE.person details.v1
- openEHR-DEMOGRAPHIC-CLUSTER.person_identifier.v1
- openEHR-DEMOGRAPHIC-PARTY_IDENTITY.person_name.v1
- openEHR-EHR-INSTRUCTION.request-imaging_exam.v1
- openEHR-EHR-OBSERVATION.imaging_exam.v1
- openEHR-EHR-INSTRUCTION.request-lab_test.v1
- openEHR-EHR-OBSERVATION.lab_test.v1
- openEHR-EHR-OBSERVATION.lab_test-blood_gases.v1
- openEHR-EHR-OBSERVATION.lab_test-full_blood_count.v1
- openEHR-EHR-OBSERVATION.lab_test-liver_function.v1
- openEHR-EHR-OBSERVATION.lab_test-thyroid.v1
- openEHR-EHR-OBSERVATION.lab_test-urea_and_electrolytes.v1
- openEHR-EHR-CLUSTER.lab_result_annotation.v1
- openEHR-EHR-CLUSTER.organisation.v1
- openEHR-EHR-CLUSTER.person_name.v1

One archetype is newly designed:

- openEHR-EHR-OBSERVATION.lab_test-general.v1

**Fig. 3:**
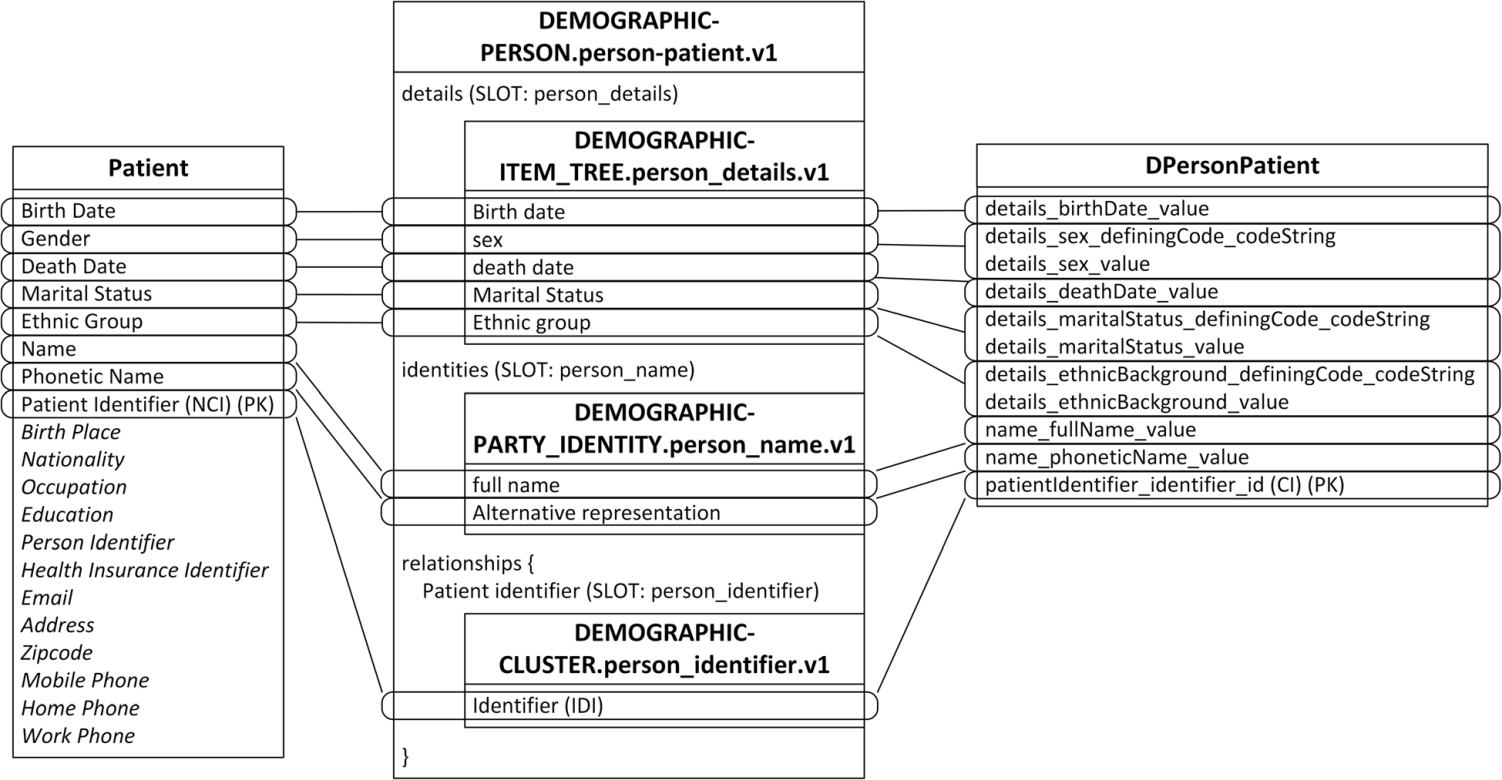
Mapping of openEHR-DEMOGRAPHIC-PERSON.person-patient.v1. In all figures, PK stands for primary key. FK stands for foreign key, and indicates the data item which current data item relates to. SLOT indicates target archetype which conform to current data item. Solid line indicates foreign key relationship between archetypes. Dash line indicates composition relationship through slot between archetypes. IDI stands for identification data item. QDI stands for query data item. CI stands for clustered indexed. NCI stands for non-clustered indexed. Data items in italic type are not covered by archetypes

**Fig. 4:**
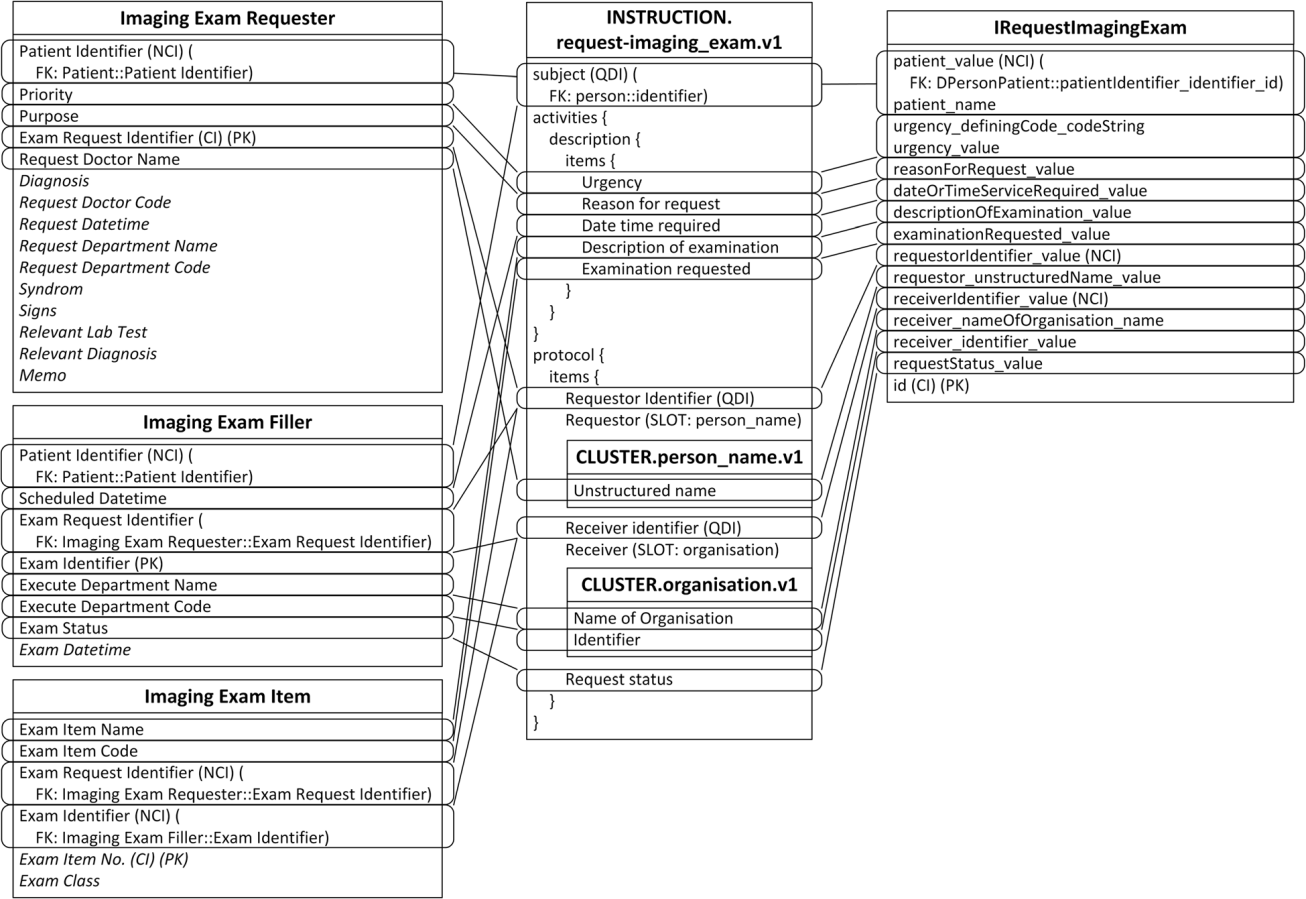
Mapping of openEHR-EHR-INSTRUCTION.request-imaging_exam.v1. In all figures, PK stands for primary key. FK stands for foreign key, and indicates the data item which current data item relates to. SLOT indicates target archetype which conform to current data item. Solid line indicates foreign key relationship between archetypes. Dash line indicates composition relationship through slot between archetypes. IDI stands for identification data item. QDI stands for query data item. CI stands for clustered indexed. NCI stands for non-clustered indexed. Data items in italic type are not covered by archetypes

**Fig. 5:**
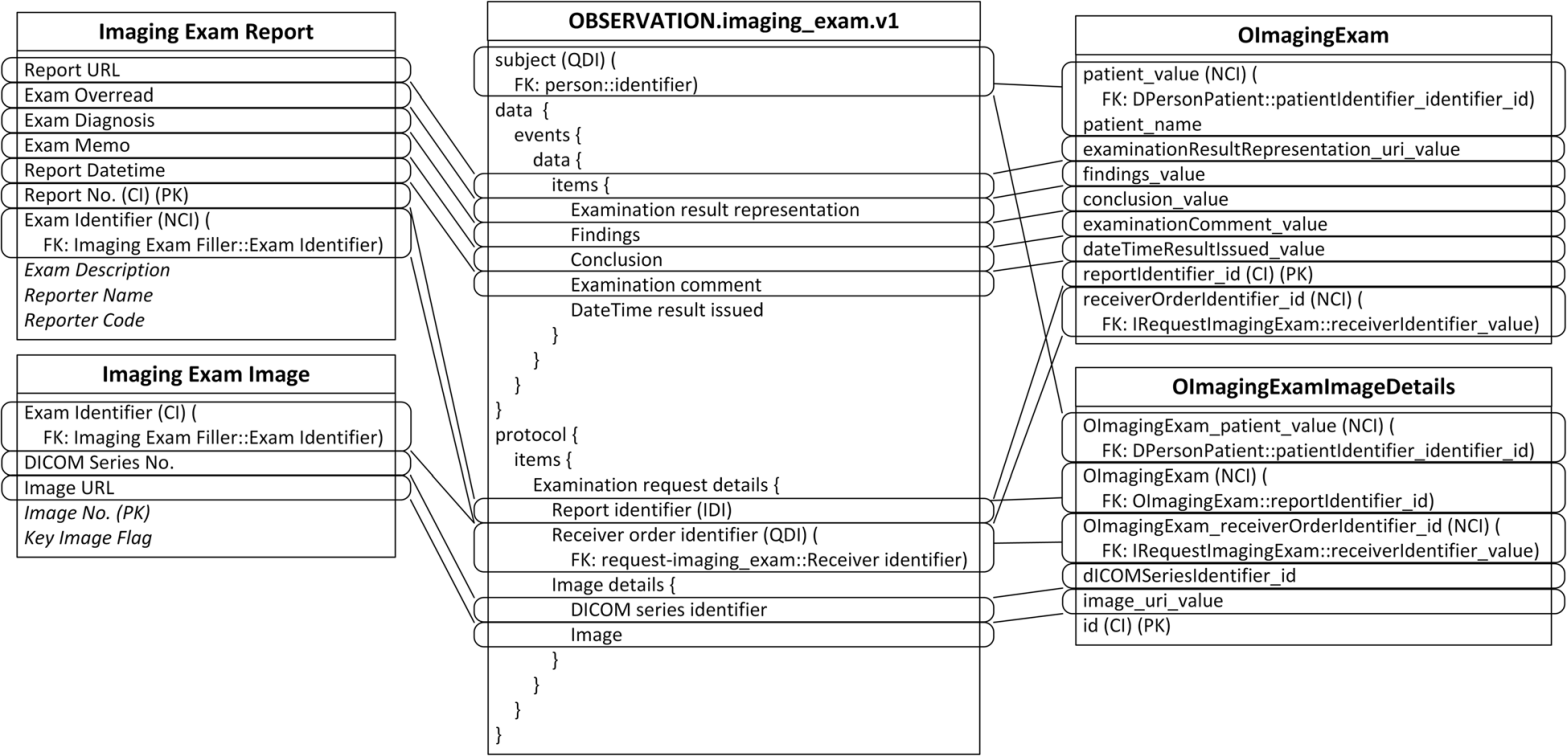
Mapping of openEHR-EHR-OBSERVATION.imaging_exam.v1. In all figures, PK stands for primary key. FK stands for foreign key, and indicates the data item which current data item relates to. SLOT indicates target archetype which conform to current data item. Solid line indicates foreign key relationship between archetypes. Dash line indicates composition relationship through slot between archetypes. IDI stands for identification data item. QDI stands for query data item. CI stands for clustered indexed. NCI stands for non-clustered indexed. Data items in italic type are not covered by archetypes

Due to the different granularity and reusability between archetypes and IV concepts, data items belonging to one IV concept are commonly scattered into several archetypes, and vice versa. For example, the Patient concept is mapped to four archetypes, represented by slots in Fig. 3. The archetype openEHR-EHR-INSTRUCTION.request-imaging_exam.v1 is mapped to three IV concepts, as shown in Fig. 4, and the archetype openEHR-EHR-OBSERVATION.imaging_exam.v1 is mapped to two IV concepts, as shown in Fig. 5.

**Fig. 6:**
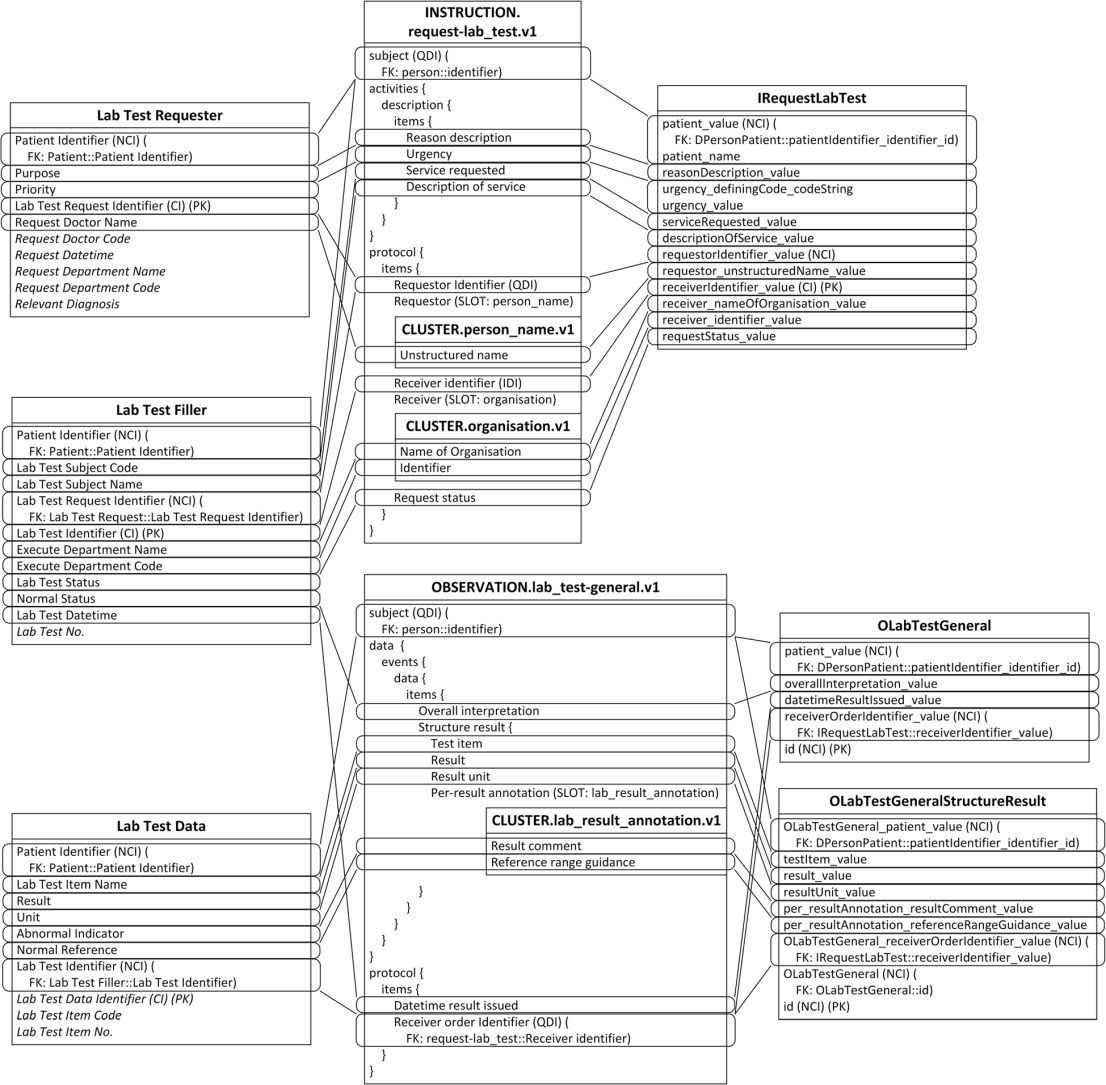
Mapping of openEHR-EHR-INSTRUCTION.request-lab_test.v1 and openEHR-EHR-OBSERVATION.lab_test-general.v1. In all figures, PK stands for primary key. FK stands for foreign key, and indicates the data item which current data item relates to. SLOT indicates target archetype which conform to current data item. Solid line indicates foreign key relationship between archetypes. Dash line indicates composition relationship through slot between archetypes. IDI stands for identification data item. QDI stands for query data item. CI stands for clustered indexed. NCI stands for non-clustered indexed. Data items in italic type are not covered by archetypes

Another common situation is a distinction between metadata-level modelling versus data-level modelling [29]. For example, there are many specific lab test result archetypes, such as blood gases, full blood count, liver function, etc., while the number of lab test results in IV is greater than 200; additionally, more results will be generated with new technologies and instruments. Since the lab test result items all exhibit a similar data structure, it is convenient to define a generalized archetype openEHR-EHR-OBSERVATION.lab_test-general.v1 specialized from archetype openEHR-EHR-OBSERVATION.lab_test.v1 with three additional multiple occurrence data items (Test Item, Result, and Result Unit) according to the Lab Test Data concept in IV (Fig. 6), along with the specialized archetypes to represent these flexible lab test results.

The subject data item in every archetype is used to represent the patient himself.

Table 3 lists the templates and ARM constraints defined according to IV data requirements.

**Table 3:** Templates and ARM constrains

Template	Identification data item	Query data item
PERSON.person-patient.v1.oet	patientIdentifier_identifier_id	(NONE)
INSTRUCTION.request-imaging_exam.v1.oet	id	requestorIdentifier_value
		patient_value
		receiverIdentifier_value
INSTRUCTION.request-lab_test.v1.oet	receiverIdentifier_value	requestorIdentifier_value
		patient_value
OBSERVATION.imaging_exam.v1.oet	id	receiverOrderIdentifier_id
		patient_value
OBSERVATION.lab_test-general.v1.oet	id	receiverOrderIdentifier_value
		patient_value

Mappings between the generalized archetype lab_test-general.v1 and specialized archetypes lab_test-blood_gases.v1, lab_test-full_blood_count.v1, lab_test-liver_function.v1, lab_test-thyroid.v1, and lab_test-urea_and_electrolytes.v1 are also defined. For example, as shown in Table 4, name, value, and unit of data item White Cell Count in openEHR-EHR-OBSERVATION.lab_test-full_blood_count. v1 are mapped to three data items (Test Item, Result, and Result Unit) in openE HR-EHR-OBSERVATION.lab_test-general.v1.

**Table 4:** Template file of openEHR-EHR-OBSERVATION.lab_test-full_blood_count.v1

<eav name = “openEHR-EHR-OBSERVATION.lab_test-general.v1” > </eav>
<eavAttributeName name = “[White cell count]” set = “en”>
[Test item]/value/value
</eavAttributeName>
<eavAttributeField name = “[White cell count]/value/magnitude”>
[Result]/value/value
</eavAttributeField>
<eavAttributeField name = “[White cell count]/value/units”>
[Result unit]/value/value
</eavAttributeField>

The < eav > node indicates the target generalized archetype to which current specialized archetype is mapped. In the < eavAttributeName > node, the attribute “name” specified the full path of the source data item, the attribute “set” indicates which textual name provided within the archetype ontology section is used since there are multi languages, and the value is the full path of the target data item. In the < eavAttributeField > node, the attribute “name” specified the full path of one data field in the source data item and the value is the full path of the target data item

**Fig. 7:**
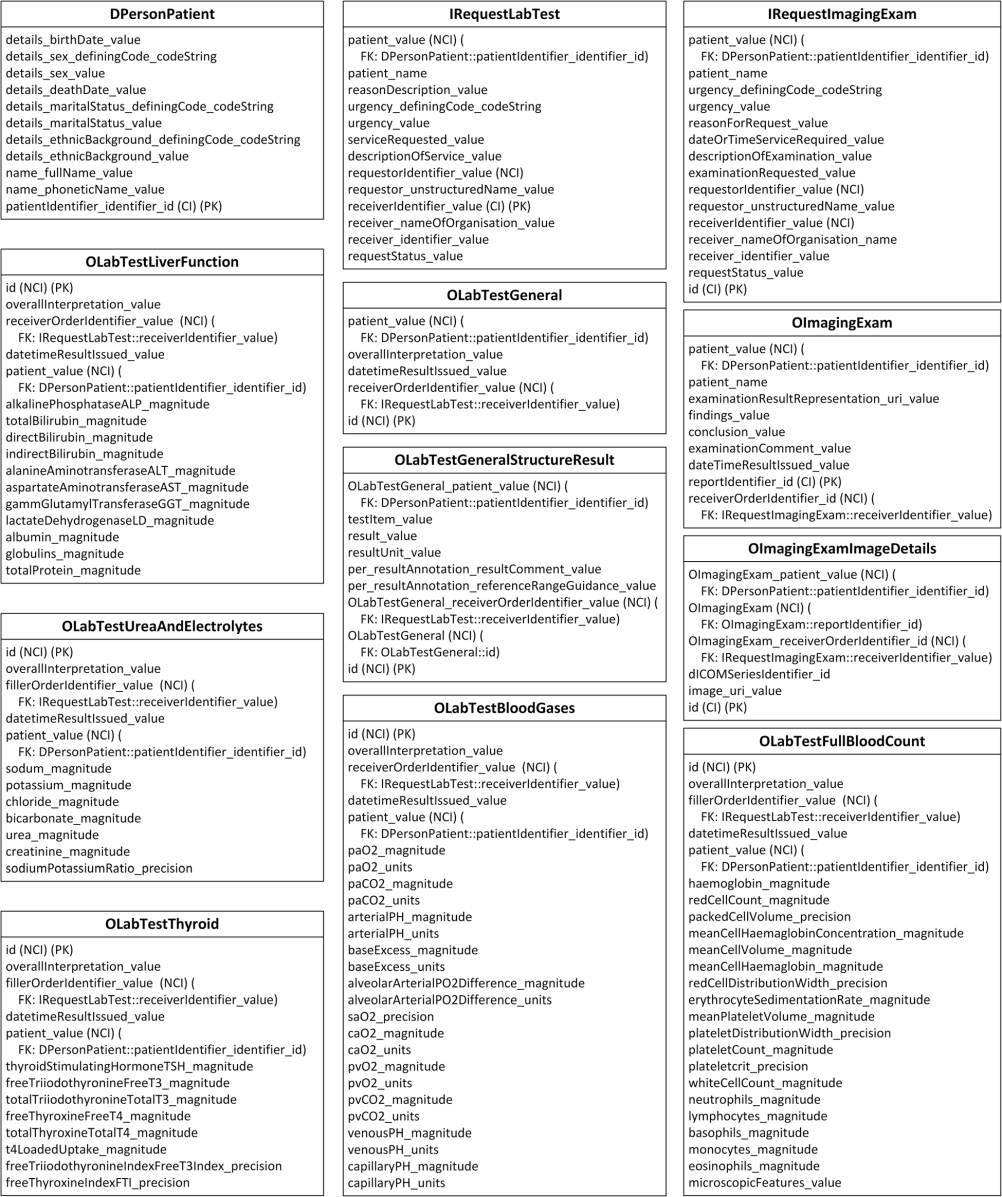
ARM database schema. In all figures, PK stands for primary key. FK stands for foreign key, and indicates the data item which current data item relates to. SLOT indicates target archetype which conform to current data item. Solid line indicates foreign key relationship between archetypes. Dash line indicates composition relationship through slot between archetypes. IDI stands for identification data item. QDI stands for query data item. CI stands for clustered indexed. NCI stands for non-clustered indexed. Data items in italic type are not covered by archetypes

Finally, the ARM mapping rules are applied to the archetypes, templates, and ARM constraints to generate the final relational database schema, as shown in Fig. 7.

### Database preparation

To determine whether the performance can meet the requirements of clinical practice, a performance comparison is conducted between the generated ARM database, conventional IV database, and the official Node + Path database.

**Fig. 8:**
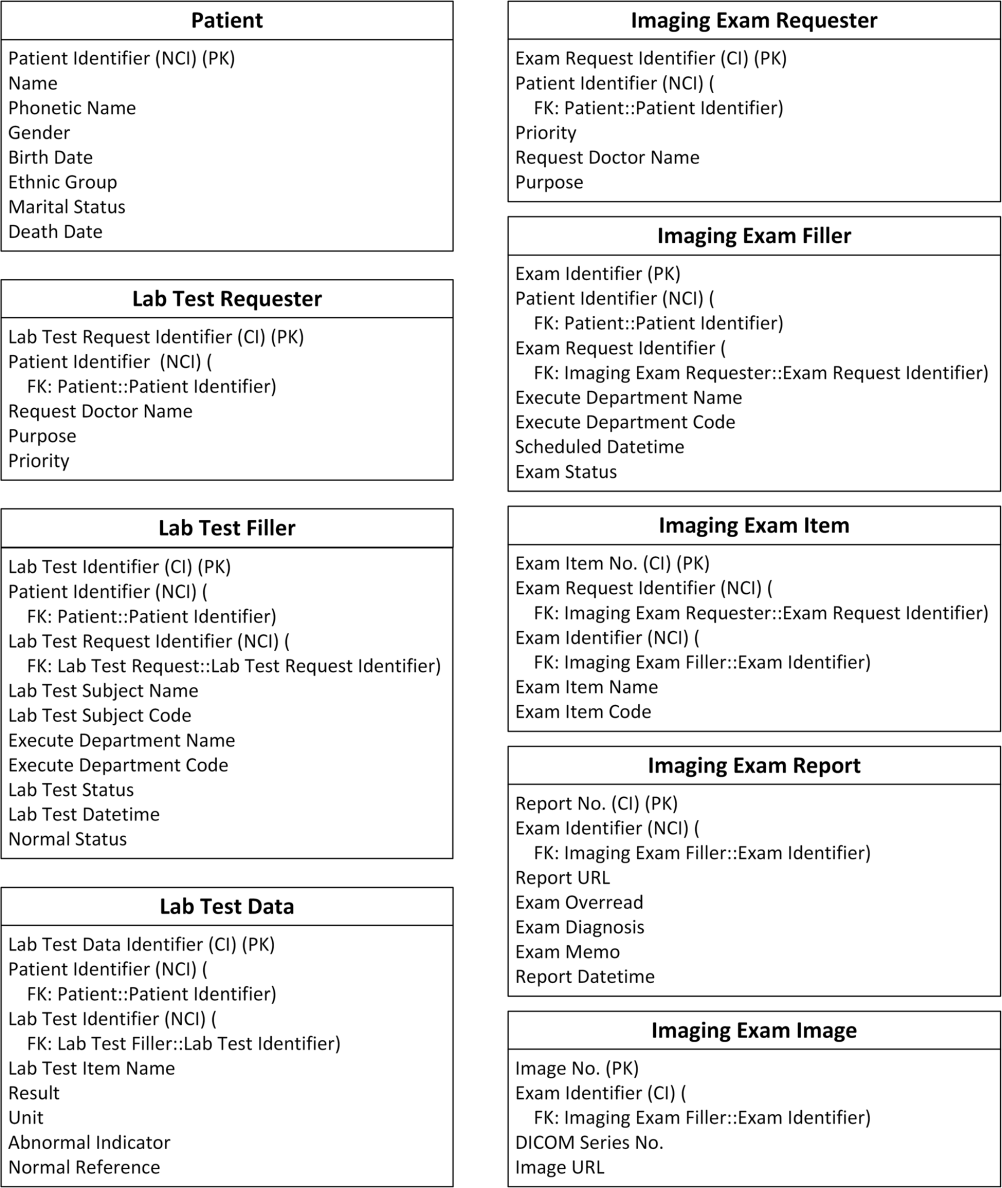
Test IV database schema. In all figures, PK stands for primary key. FK stands for foreign key, and indicates the data item which current data item relates to. SLOT indicates target archetype which conform to current data item. Solid line indicates foreign key relationship between archetypes. Dash line indicates composition relationship through slot between archetypes. IDI stands for identification data item. QDI stands for query data item. CI stands for clustered indexed. NCI stands for non-clustered indexed. Data items in italic type are not covered by archetypes

**Fig. 9:**
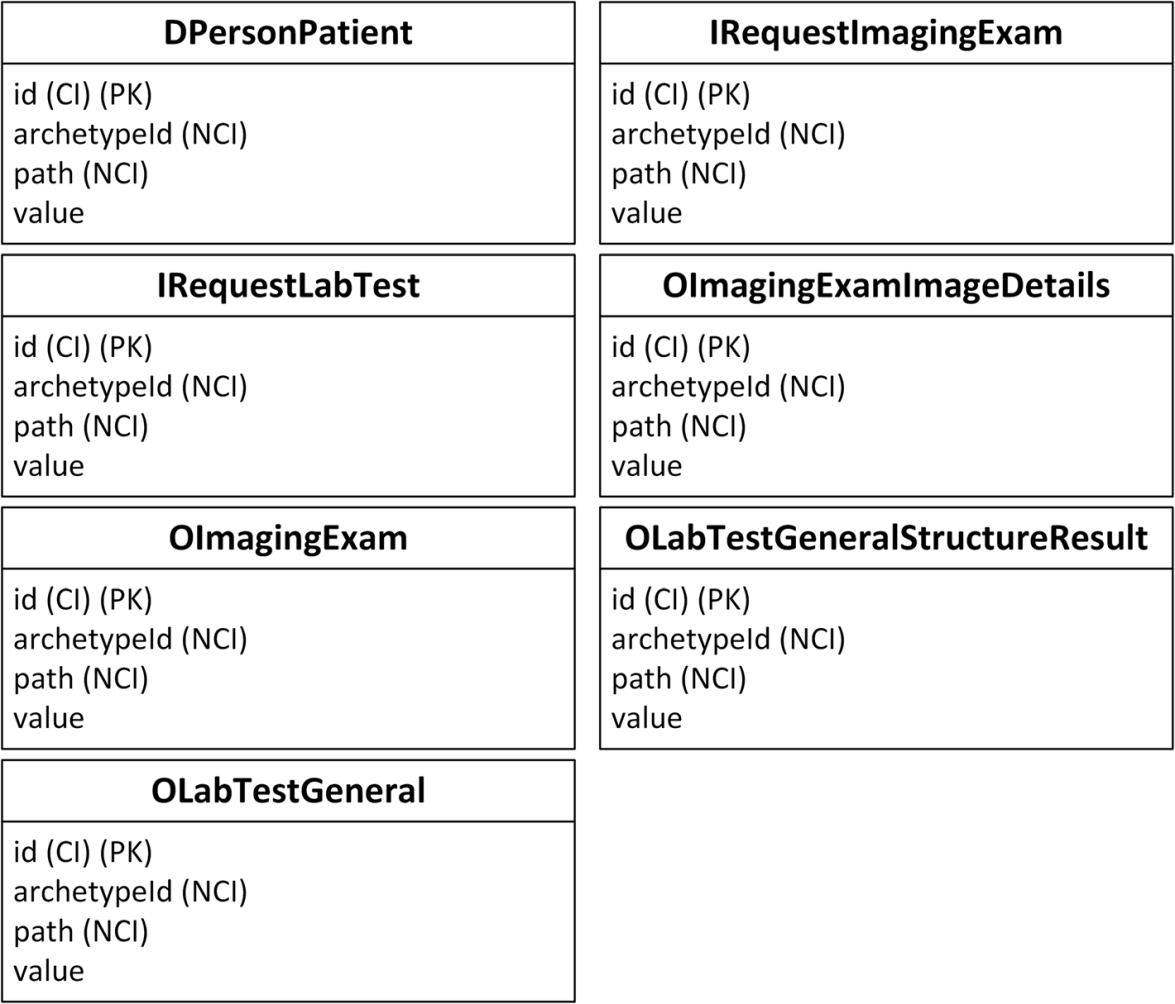
Node + Path database schema. In all figures, PK stands for primary key. FK stands for foreign key, and indicates the data item which current data item relates to. SLOT indicates target archetype which conform to current data item. Solid line indicates foreign key relationship between archetypes. Dash line indicates composition relationship through slot between archetypes. IDI stands for identification data item. QDI stands for query data item. CI stands for clustered indexed. NCI stands for non-clustered indexed. Data items in italic type are not covered by archetypes

The schema of the test IV database is shown in Fig. 8. Data items not encompassed by archetypes are removed to maintain comparability of the test IV database. The Node + Path database schema is shown in Fig. 9. Although only one table is required to store all data, one table is assigned to each concept to promote practicality and greatly improve performance.

A dataset is extracted directly from the online IV database, with dates ranging from 2014-01-01 to 2014-12-31, and containing 103320 imaging tests, 8573157 images, 654213 laboratory tests, and 4846688 laboratory test result items for 29743 patients. All data has been de-identified by removing all patient names, patient phonetic names, patient birth dates, patient death dates, and doctor names. The dataset is imported into three clean instances of the test IV database, the ARM database, and the Node + Path database. The IV database requires 1.60 gigabytes on the hard disk, the ARM database requires 2.90 gigabytes, and the Node + Path database requires 43.87 gigabytes, which is far greater than the space required by the other two databases. Although the Node + Path is much more efficient in storing sparse data, it also includes too many redundancies to store the path of each archetype data item.

### Query benchmark

Tests are conducted on a Dell M4700 running WINDOWS 8.1 Enterprise 64 bit operating system and Microsoft SQL Server 2014 Enterprise Edition with an Intel Core i5-3340 M processor, 16 gigabytes of memory, and a 5400-RPM hard disk.

Clinicians use the IV each day to monitor patient imaging exams and laboratory tests in order to make further decisions. The IV presents a patient list for each clinician; when a patient is selected, all correlated imaging exams and laboratory tests are displayed in two pages, enabling the clinician to click on each imaging exam or laboratory test to verify all the results, images, and reports in detail. The IV updates all information in real time to support clinician responses to patient situations with minimal time delays.

Five data-retrieving tests are designed from this workflow scenario:

- Test 1: Find all patients belonging to a single clinician to generate a daily work list. A clinician sees at least 1 patient per day (Query 1.1), an average of 7 patients per day (Query 1.2), and a maximum of 50 patients per day (Query 1.3).
- Test 2: Find all imaging exams for a single patient. A patient has at least 1 imaging exam (Query 2.1), an average of 3 imaging exams (Query 2.2), and a maximum of 26 imaging exams (Query 2.3).
- Test 3: Find all images related to a single imaging exam. An imaging exam contains at least 1 image (Query 3.1), an average of 363 images (Query 3.2), and a maximum of 10664 images (Query 3.3).
- Test 4: Find all laboratory tests for a single patient. A patient has at least 1 laboratory test (Query 4.1), an average of 23 laboratory tests (Query 4.2), and a maximum of 425 laboratory tests (Query 4.3).
- Test 5: Find all results in a single laboratory test. A laboratory test contains at least 1 result (Query 5.1), an average of 168 results (Query 5.2), and a maximum of 4477 results (Query 5.3).

In addition to these five data-retrieving tests, two patient-searching tests are designed to test performance in finding patients who satisfy certain criteria. Finding similar patients is integral to evidence-based care delivery, and helps clinicians make further decisions. However, because IV defines many concepts in the data level, it is not efficient to implement this with the conventionally-designed IV database. However, with the archetype approach, concepts are explicitly expressed as archetypes and can be mapped to standalone tables.

- Test 6: Find all patients with PaO2 > = 129 mmHg in blood gas tests (Query 6.1). Find all patients with PaO2 > = 129 mmHg, PaCO2 > = 27 mmHg, and Arterial pH > = 7.3 in blood gas tests (Query 6.2). Find all patients having abnormal PaO2 > = 129 mmHg, PaCO2 > = 27 mmHg, Arterial pH > = 7.3, SaO2 > = 99 %, and CaO2 > = 17 % value in blood gas tests (Query 6.3).
- Test 7: Find all patients with PaO2 > = 229 mmHg in blood gas tests, red cell count > = 2 1012/L in full blood count tests, and alkaline phosphatase > = 50 IU/L in liver function tests (Query 7.1). Find all patients with PaO2 > = 229 mmHg in blood gas tests, red cell count > = 2 1012/L in full blood count tests, alkaline phosphatase > = 50 IU/L in liver function tests, thyroid stimulating hormone > = 0.3 μIU/mL in thyroid tests, and sodium > = 140 mmol/L in urea-electrolyte tests (Query 7.2).

Table 5 lists the benchmark results of test queries for each database. All queries are composed of multiple, simple SQL clauses to avoid joining tables or clause nesting, resulting in better performance according to clinical practice. Each query was executed ten times, and the average time was calculated. The database cache is turned off to avoid the caching effects of the selected database product.

**Table 5:** Query benchmark

Query	IV (ms)	ARM (ms)	Node + Path (ms)
Query 1.1	80 (+74 %)	46	5017
Query 1.2	91 (+54 %)	59	5121
Query 1.3	196 (+15 %)	170	5358
Query 2.1	221 (+16 %)	191	24866
Query 2.2	219 (+17 %)	187	25094
Query 2.3	474 (+129 %)	207	26158
Query 3.1	242	270 (+12 %)	294774
Query 3.2	224	299 (+33 %)	297388
Query 3.3	254	411 (+62 %)	362950
Query 4.1	198 (+13 %)	176	127547
Query 4.2	254 (+32 %)	193	128508
Query 4.3	1249 (+57 %)	797	129901
Query 5.1	113	186 (+65 %)	328181
Query 5.2	125	205 (+64 %)	329097
Query 5.3	139	239 (+72 %)	388727
Query 6.1	14596 (+5150 %)	278	5746
Query 6.2	16340 (+5293 %)	303	6029
Query 6.3	16453 (+5140 %)	314	6984
Query 7.1	14582 (+1028 %)	1293	41217
Query 7.2	14649 (+669 %)	1904	53352

The percentage values in IV and ARM columns are more time spent on each query in the slower database than the faster database. The Node + Path database is not included in the calculation

The performances of the ARM database and IV database were very similar in the execution of data-retrieving tests. ARM performed better in tests 1, 2 and 4, while IV performed better in tests 3 and 5. The detailed reasoning for differences in absolute execution time is highly complex due to the nature of the complicated systems that are affected by many external factors, such as background tasks on the Windows operating system, hard disk cache, etc. There are also some database factors that contribute to the differences in execution time between the ARM database and the IV database.

In test 1, both the ARM and IV database were queried with one SQL clause, using patient id as a condition. The patientIdentifier_identifier_id column of the DPersonPatient table in the ARM database was clustered indexed, while the Patient Identifier column of the Patient table in the IV database was non-clustered indexed, which requires additional key lookup operations and thus requires more time.

In test 2, table IRequestImagingExam in the ARM database was queried using one SQL clause; however, in the IV database, three corresponding tables (Imaging Exam Requester, Imaging Exam Filler, and Imaging Exam Item) must be queried with three SQL clauses.

In test 3, two tables (OImagingExam and OImagingExamImageDetails) were queried in the ARM database, and three tables (Imaging Exam Filler, Imaging Exam Report, and Imaging Exam Image) were queried in the IV database. However, the OImagingExamImageDetails table containing 8573157 image records in the ARM database is non-clustered indexed, resulting in extra key lookup operations that are slower than the corresponding Imaging Exam Image table in the IV database, which is clustered indexed.

In test 4, one table (IRequestLabTest) in the ARM database was queried while two tables (Lab Test Requester and Lab Test Filler) must be queried in the IV database.

In test 5, both the ARM and IV databases were queried on one table with one SQL clause, using patient id as a condition. The OLabTestGeneral_patient_value column of the OLabTestGeneralStructureResult table in the ARM database is non-clustered indexed, while the Patient Identifier column of the Lab Test Data table in IV database is clustered indexed.

The Node + Path database requires more time for all tests, even when querying for few results, due to the inevitable full table scan; thus, it is not practical in a clinical workflow. All of the ARM, IV, and Node + Path databases have similar trends: as the query returns more data, the test requires more time to execute.

In patient-searching tests, the series of lab test result archetypes were directly mapped into standalone tables in the ARM database and the Node + Path database. Each table stores only data related to the corresponding archetype, in which the number of records dramatically decreases. However, in the IV database, all lab test result data are stored in an EAV-style table.

In test 6, only one table is queried with different conditions, namely: table OLabTestBloodGases in the ARM database, table Lab Test Data in the IV database, and table OLabTestBloodGases in the Node + Path database, so the increase of query time is trivial among all three databases. The ARM database was the fastest, and the IV database was slower than the Node + Path database, since it contains many more records.

In test 7, the query time increased greatly for the ARM database and the Node + Path database, in which more than one table was queried, namely tables OLabTestBloodGases, OLabTestFullBloodCount, OLabTestLiverFunction, OLabTestThyroid, and OLabTestUreaAndElectrolytes in both databases. However, only one table (Lab Test Data) was queried in the IV database. The performance of the Node + Path database was even slower than that of the IV database, since the lab test results table in the IV database is not purely EAV and thus performs much better.

## Discussion

This paper presents an ARM persistence solution for archetype-based EHR systems. While the ARM approach is designed to generate a relational database from archetypes and templates and can achieve performance similar to a conventionally-designed database, there were several encountered challenges and issues.

### ARM deployment

ARM employs a model-driven approach to allow data persistence to adapt to changes in data requirements according to archetypes that represent general domain concepts and templates tailored to ARM constraints. The mapping rules are implemented in a persistent service to automatically generate the database, and avoid necessary manual uploading of the database. Currently, the changes described in archetype semantic relationships are easy to implement on the relational database, but one change is not explicitly included in the new versions of the archetypes. In ideal archetypes, the data type of data items should be as stable as the data items, and remain unchanged. However, this cannot be avoided during archetype development, particularly for archetypes that are initially developed from local data requirements and later extended to a global scope, which often results in incompatible versions of archetypes.

Changing the column data type can induce chaos into the relational database; thus, two mapping rules are designed to automatically adapt to change. One rule maps each version of an archetype to a table; old versions of an archetype will gradually become outdated and obsolete, and can then be safely moved to a backup database. Prior to removal, multiple versions of an archetype that are simultaneously in service result in a large number of tables. The second rule maps all versions of an archetype to a new table, then imports all data from the old table into the new one according to the conversion algorithm provided with the archetype [30]; the old table is then removed. However, the data conversion process is costly in terms of time and computation if the table contains a great number of records. Although it is safe to redeploy changed archetypes and templates to update a deployed database, the principles of archetype design recommend that archetypes and templates be maintained by a committee of domain experts, and deployed when they are stable. Archetypes should be reused as much as possible to represent the domain concepts. Templates are used to align the data persistence to different data requirements, and to avoid shifting the heterogeneity of data requirements to archetypes.

### De-normalization

In archetype modelling, the most important archetype resource is the CKM, in which archetypes are maintained by healthcare experts and published in a central repository. Archetypes in CKM are highly abstract and normalized in order that each archetype represents a complete domain concept. They are revised by various experts according to various kinds of data requirements. In conventionally-designed databases, a combination of well-organized tables, tolerable redundancies for de-normalization, and fine-tuned indices allow all queries to be implemented with as few SQL clauses as possible. Several de-normalizations are introduced in ARM to achieve better performance.

First, the de-normalization of the granularity of archetypes is achieved by embedding archetypes by archetype slot mapping. Since the granularities of archetypes and data requirements are not always identical, archetypes composed by archetype slots with a single occurrence represent one concept, and can be embedded together in data requirements. In this manner, query steps can be reduced and the joining of tables can be avoided. However, the embedded archetypes are then deemed to be in a “division” state, indicating that one archetype can be slotted and embedded into many different archetypes or simultaneously used alone. The division caused by archetype de-normalization introduces further complexity to data query using the embedded archetypes. Archetypes used only as components of other concepts, such as openEHR-EHR-CLUSTER.person_name.v1, are seldom used to query data alone. For archetypes, both those mapped standalone and embedded into other archetypes, one must decide, whether to query only the standalone mapped data tables or to query all data tables containing the archetypes according to the semantics of the archetypes.

Second, de-normalization of the index redundancy of query data items propagation is achieved. Proper indices can greatly improve the data query performance of relational databases. However, it is inconvenient to add indices in archetypes, due to their high normalization. For example, to retrieve the images of a specific exam request, first has the particular exam request must be queried with the request order id, then the exam items contained in this exam request must be queried, and finally the images of each exam item must be queried. By introducing index propagation, indices can propagate through archetype slots in order to reduce the number of query steps. However, not all propagated indices are necessary to the target archetypes, and will consume space and computation time in order to be updated. These must therefore be manually configured in order to disregard unnecessary propagated indices in ARM constraints, according to the data requirements.

### Meta-data level and data-level mapping

Archetypes maintained by domain experts as common knowledge in the centralized approach will gradually accumulate value. Archetypes representing domain concepts at the meta-data level will greatly facilitate the use of clinical data, and improve the performance of data retrieval as compared to concepts defined at the data-level. As more archetypes are committed to encompass more domain concepts, it will become easier for clinicians to manipulate clinical data. For example, clinicians can use red cell count in openEHR-EHR-OBSERVATION.lab_test-full_blood_count.v1 as conditions to query the patient directly; data-retrieving performance will improve because the result data of each laboratory test are stored separately. The ARM approach reduces the manual updating process for data persistence according to changes in domain concepts, and encourages the use of meta-level data instead of data-level model methods. However, the meta-data level model lacks the universal flexibility of the data-level model. In order to adapt to evolving requirements, the meta-data level model must first define archetypes, and then generate data persistence while the generic structure of the data-level model does not change. In ARM, the meta-data level model and the data-level model are combined to utilize the advantageous of both approaches. The archetype openEHR-EHR-OBSERVATION.lab_test-general.v1 is defined to improve flexibility, and specific archetypes are introduced to facilitate the data query.

### Limitations of ARM

Although the ARM approach can provide similar performance to the conventional database, it may not meet the requirements of situations in which the databases must be highly-tuned. For example, there are no one-size-fits-all rules of indices; they must be adjusted according to queries or even internal data distribution in the databases. The ARM strikes a balance between automation and performance to generate data persistence with archetypes.

If the local data requirements can be properly satisfied, the published archetypes are of great value to ARM. When the hierarchy and structure of archetypes are similar to the data requirements, few reorganizations are necessary before application of the ARM approach. If the archetypes and data requirements exhibit structural differences, many reconfigurations are necessary to align the archetypes to the data requirements, possibly including extensions and modifications to the archetypes. However, published archetypes are limited in scope compared to the enormous amount of clinical concepts. New archetypes must be developed if local data requirements are not satisfied. The process of archetype development is very restrictive and requires extensive professional knowledge in order to develop and model high-quality archetypes. This will require much more effort than the conventional database design approach.

### Affections to archetypes and templates

In general, the ARM approach conforms to the design principles of archetypes and templates. For example, each archetype should represent a single concept. This represents best practice in database normalization, and the composition of archetypes into large templates is introduced via the concept of slot embedment. Furthermore, the ARM places more emphasis on subtle details in order to achieve better design of archetypes and templates. For instance, ARM requires related data items within archetypes to be organized as clusters in order to explicitly express their relationships. If two data items both have multiple occurrences and a one-to-one relationship, they must be altered to a single occurrence and put into a multiple occurrence cluster; this cluster can then be moved into a standalone cluster archetype and reused elsewhere. In template design, ARM requires the identification data item and query data items to add indices. Although it can be difficult to assign the correct roles to the correct data items, they can help the designer achieve better understanding of the function of each data item when the templates are used as data entry documents, graphical user interface models, or data-retrieving queries. ARM refines the design principles of archetypes and templates by considering their practical use and application in clinical practice.

## Conclusions

This paper presents an ARM persistence solution for archetype-based EHR systems. ARM uses archetypes to generate relational databases and achieve similar performance as compared to conventional databases in data-retrieving queries. ARM takes great advantage of the CKM public archetype repository to facilitate data manipulation with well-defined archetypes for clinicians and to achieve better performance in patient-searching queries. System components like ARM can facilitate the adoption of openEHR architecture in EHR systems. The authors will continue to complete the mapping rules according to the semantics of archetypes, improve ARM constraints and the performance of the generated databases, design and implement data access services, and perform thorough tests of the ARM approach in real clinical environments.

## Abbreviations

EHR: Electronic health record
ARM: Archetype relational mapping
CKM: Clinical Knowledge Manager
RM: Reference model
EAV: Entity-attribute-value
ORM: Object Relational Mapping
RIM: Reference Information Model
HMIS: Hospital management information system
LIS: Laboratory information system
RIS: Radiology information system
PACS: Picture archiving and communication system
PMS: Pharmacy management system
OMS: Operation management system
CPOE: Computerized Physician Order Entry
IV: Integrated Viewer
