# Erratum to: Archetype relational mapping - a practical openEHR persistence solution

**DOI:** 10.1186/s12911-016-0259-6

**Published:** 2016-02-12

**Authors:** Li Wang, Lingtong Min, Rui Wang, Xudong Lu, Huilong Duan

**Affiliations:** College of Biomedical Engineering and Instrument Science, Zhejiang University, Room 512, Zhouyiqing Building, Zhejiang University, 38 Zheda Road, Hangzhou, Zhejiang China; Department of Information Technology, Shanxi Dayi Hospital, Taiyuan, Shanxi China

Following the publication of the original article [1], it was brought to our attention that citation number 20 had been truncated. At current the reference reads as:

‘20. Freire SM, Sundvall E, Karlsson D, Lambrix P. Performance of XML Databases for Epidemiological Queries in Archetype-Based EHRs. 2012:51–57.’

However, it should instead read as:

‘20. Freire SM, Sundvall E, Karlsson D, Lambrix P. Performance of XML Databases for Epidemiological Queries in Archetype-Based EHRs. Scandinavian Conference on Health Informatics 2012:51–57. http://www.ep.liu.se/ecp/070/009/ecp1270009.pdf’
